# Supplementary material for: Biochemical, Clinical, and Genetic Characteristics of Mexican Patients with Primary Hypertriglyceridemia, Including the First Case of Hyperchylomicronemia Syndrome Due to GPIHBP1 Deficiency
Source: Int J Mol Sci. 2022 Dec 27;24(1):465. doi: 10.3390/ijms24010465 (PMC9820378; doi:10.3390/ijms24010465)
Supplement: Supplementary file 1 [file ijms-24-00465-s001.zip › Suplementary Table S2.pdf]

Supplementary Table S2. Primers used to amplify the 30 segments studied in this research

Exon	Sequence 5' to 3'	Length (bp)
APOA5		
1-F<br>1-R	CAGTTGCCCCTGCAGAGG<br>CTCCCCCAGGGTGGACAG	231
2-F<br>2-R	TTCAACGTGGGGGTGTGG<br>AGGCAGCAGAGGCAGGTC	285
3A-F[a]<br>3A-R	TCAGTGCGCGATGACTTG<br>GGCCACCTGCTCCATCAG	342
3B-F<br>3B-R	TGCGGCAGCAACTGAAGC<br>ACCTGCACGCAGCGACTG	316
3C-F<br>3C-R	GAGCTGCACCGCAGTGTG<br>GCTGCTGCTGGACCTCCTC	319
3D-F<br>3D-R	CGCCAGGACACCTACCTGC<br>CTTCCACCCTCCACCCAAC	346
GPIHBP1		
1-F<br>1-R	GCTGGGGACCAGTGTGAG<br>TCTTCCTAAGCCCCTGCTG	295
2-F<br>2-R	CACGATGCTTGCCCAGAG<br>CCTGCTGGCTTCCATCAC	285
3-F<br>3-R	GAACGGGGAGGTGGACAG<br>GGCGGGGTCTCTGAGGTG	287
4-F<br>4-R	AACAGAGCCCTGCAGAGCCAC<br>TCCAAATCCATTCTCCAAAGCTG	438
LMF1		
1-F<br>1-R	TCAGCTGTGAGGCAGGGGCAC<br>TAGCCCCGAGCCGGTCCTGG	932
2-F<br>2-R	AGACTCTGCCTCCCCTTC<br>CCAGTCTTTCCAAGTGCC	510
3-F<br>3-R	CAGGTCCTCCTCGCAGGG<br>AAGGCTGATGGCAGAGGC	262
4-F<br>4-R	CAGCCCTGTCTCAAGGAC<br>GGGGGAGGAAGGAAACAG	353
5-F<br>5-R	CCTTCGTGGATGGTTCGTC<br>CCAGGGTGTGCAGGACTG	255
6-F<br>6-R	GGACCCACAAGCCTCTCCCTAG<br>CTGTAATGCCCCAGGGTCCTC	344
7-F<br>7-R	CCTCCCTGCTCCAGGAAGAGAG<br>CCTTGTTCCTGGCACGCTACAC	431

8-F<br>8-R	ACCCTTAGGGAGCAGCAGC<br>GCCTGCACTGTAACCCCAC	334
9-F<br>9-R	CTCAGTAGCCCCTGACCAGGAG<br>CTTCAGTGGGCGTTCTAGAAACC	423
10-F<br>10-R	CCCACCTCCAGGAAAGCCTCAG<br>TGCAGGTACAGGTGGTGGGATC	305
11-F<br>11-R	GTCTACCTGTGCTCAGGGTGGG<br>GCTCTCCTCTCCACGTCTCTCTTG	439
LPL		
1-F<br>1-R	GCTGCCCACTTCTAGCTG<br>CCCCTTCCAACTTCCTTC	330
2-F<br>2-R	TCAGAAACAAAAATAGCATCAG<br>GAGATCCACGTGAGATGTG	389
3-F<br>3-R	CATTTCATGCAGGTGTATTG<br>AAAGAACAGCCGGTTTTC	338
4-F<br>4-R	TTTCAGTATTTCCTATATTTGG<br>CACACATGTGGGTATTTAAC	316
5-F<br>5-R	TCAAATGATGAGCAGTGAC<br>CTGAGTAGGACATTGGGTC	396
6-F<br>6-R	TCACCTATTTTAGACATGCC<br>GTACATGTGATGCAGTGAGC	397
7-F<br>7-R	TGAATTGCCTGACTATTTGG<br>CTCTCTGCTTCTGCTGCTC	278
8-F<br>8-R	GTGCATTTTTAAAATATCCCC<br>CTCCCTGAATTGTGAAGG	397
9-F<br>9-R	TATCAAAACAATTACCCAGC<br>ACTGATTGAATGCTTTGATC	356
F: forward, R: reverse. [a]For analysis, exon3 of the APOA5 gene was divided into four fragments		
